# Multi-genome metabolic modeling predicts functional inter-dependencies in the *Arabidopsis* root microbiome

**DOI:** 10.1186/s40168-022-01383-z

**Published:** 2022-12-09

**Authors:** Alice Mataigne, Nathan Vannier, Philippe Vandenkoornhuyse, Stéphane Hacquard

**Affiliations:** 1 Université de Rennes 1, CNRS, UMR6553 ECOBIO, Campus Beaulieu, 35000 Rennes, France; 2 Max Planck Institute for Plant Breeding Research, Department of Plant Microbe Interactions, 50829 Cologne, Germany

## Abstract

**Background:**

From a theoretical ecology point of view, microbiomes are far more complex than expected. Besides competition and competitive exclusion, cooperative microbe-microbe interactions have to be carefully considered. Metabolic dependencies among microbes likely explain co-existence in microbiota.

**Methodology:**

In this in silico study, we explored genome-scale metabolic models (GEMs) of 193 bacteria isolated from *Arabidopsis thaliana* roots. We analyzed their predicted producible metabolites under simulated nutritional constraints including “root exudate-mimicking growth media” and assessed the potential of putative metabolic exchanges of by- and end-products to avoid those constraints.

**Results:**

We found that the genome-encoded metabolic potential is quantitatively and qualitatively clustered by phylogeny, highlighting metabolic differentiation between taxonomic groups. Random, synthetic combinations of increasing numbers of strains (SynComs) indicated that the number of producible compounds by GEMs increased with average phylogenetic distance, but that most SynComs were centered around an optimal phylogenetic distance. Moreover, relatively small SynComs could reflect the capacity of the whole community due to metabolic redundancy. Inspection of 30 specific end-product metabolites (i.e., target metabolites: amino acids, vitamins, phytohormones) indicated that the majority of the strains had the genetic potential to produce almost all the targeted compounds. Their production was predicted (1) to depend on external nutritional constraints and (2) to be facilitated by nutritional constraints mimicking root exudates, suggesting nutrient availability and root exudates play a key role in determining the number of producible metabolites. An answer set programming solver enabled the identification of numerous combinations of strains predicted to depend on each other to produce these targeted compounds under severe nutritional constraints thus indicating a putative sub-community level of functional redundancy.

**Conclusions:**

This study predicts metabolic restrictions caused by available nutrients in the environment. By extension, it highlights the importance of the environment for niche potential, realization, partitioning, and overlap. Our results also suggest that metabolic dependencies and cooperation among root microbiota members compensate for environmental constraints and help maintain co-existence in complex microbial communities.

Video Abstract

**Supplementary Information:**

The online version contains supplementary material available at 10.1186/s40168-022-01383-z.

## Introduction

Microorganisms are known to colonize macroorganisms by establishing a dense network of interactions and contributing to essential functions that maintain their host homeostasis [1]⁠. These functions are varied, ranging from protection against pathogens to nutrient uptake, and resistance to stresses such as heat or drought [2, 3]⁠. Together with other factors such as temperature, pH, oxygen, nutrients [4]⁠⁠, or priority effects [5]⁠, the host itself is a niche that influences the composition of its microbiota [6, 7]⁠. In plants, soil can be considered as a reservoir of microorganisms from which microorganisms are recruited to form the root microbiota [8, 9]⁠ and where root exudates play an active role [10, 11]⁠. Last, microbe-microbe interactions are essential in shaping the structure and dynamics of microbiota [12–14]⁠, resulting in a dense network of interactions [15]⁠.

Understanding the diverse interactions between microbes is a critical step in achieving a holistic, community-level understanding of microbiota functioning. These microbe-microbe interactions exist on a spectrum from competition to cooperation [16]⁠, depending on time, space, the presence of other species, and energetic cost (etc.) [12]⁠. Two main competing theories explain microbial assemblages. The niche differentiation theory states that phylogenetically similar species are more likely to compete due to their shared functional traits and overlapping resources, leading to less probable co-existence [17]⁠. The habitat filtering theory suggests that dominant species exhibit similar functional traits because their presence is determined by environmental parameters [18]⁠. The relative importance of competition and cooperation remains unclear [16, 19]⁠. Different approaches have produced conflicting results: several computational approaches predict cross-feeding possibilities whereas several in vitro experiments rather pointed to competition [16]⁠. Nevertheless, metabolic interactions and particularly metabolic dependencies are reported to play a major role in maintaining community diversity and stability and in explaining microbial co-existence [15, 20–24]⁠. Extracellular metabolites can thus play a major role in microbial community assembly [25]⁠, and metabolic dependencies among strains may explain why some microbes cannot be cultured in standard laboratory conditions [26]⁠.

Microbial Systems Ecology is now regularly used to model complex systems such as ecological processes [27–31]. The acquisition and analysis of -omics data, coupled with modeling approaches, make it possible to computationally predict an organism’s resource use, biosynthetic capabilities, deficiencies, and growth in different conditions, notably available nutrients [26, 28]⁠, hereafter referred to as “nutritional constraints” (Table 1). These models rely on the reconstruction of metabolic networks, genome-scale metabolic models (GEMs) from annotated genomes [31, 32]⁠. Thus, making it possible to predict fundamental niche overlaps and competition between members of the same microbial community [33]⁠. Studying the phylogenetic structure of microbial communities also enables the detection of correlations between the phylogenetic signal and metabolism [34–37]⁠.

We investigated in silico how phylogeny shapes GEMs at the scale of both individual strains (1) and small random combinations of strains (SynComs) (2), tested the strength of the effect of the constraint applied by available nutrients (including root exudates) on GEMs (3) and inspected whether metabolic cooperation among strains can alleviate these nutritional constraints (4). We tested four hypotheses: (i) unconstrained metabolism (see definition in Table 1) is highly clustered by phylogeny, meaning that predicted producible metabolites are differentiated or overlap between strains according to their taxonomy (H1); (ii) combinations of GEMs have more producible metabolites than single GEMs depending on the phylogenetic similarity between the corresponding bacteria (H2); (iii) available nutrients may have an impact on the metabolism of bacteria, leading to a reduction of producible metabolites from unconstrained to constrained metabolism (H3); and (iv) metabolic cooperation is common and likely compensates for nutritional constraints by allowing the production of specific key compounds (H4) under the strong hypothesis that every compound produced by a bacterium can be shared with others. This hypothesis is at least partially supported by several studies which analyzed or predicted bacteria and plant secretomes [38–40]⁠. To test these hypotheses, we analyzed a collection of genomes of bacterial strains isolated from the roots of *Arabidopsis thaliana* [41]⁠ and used systems biology approaches to predict genome functioning in silico. The metabolism of each bacterium was predicted with GEMs reconstructed using genome annotation.

## Materials and methods

**Fig. 1:**
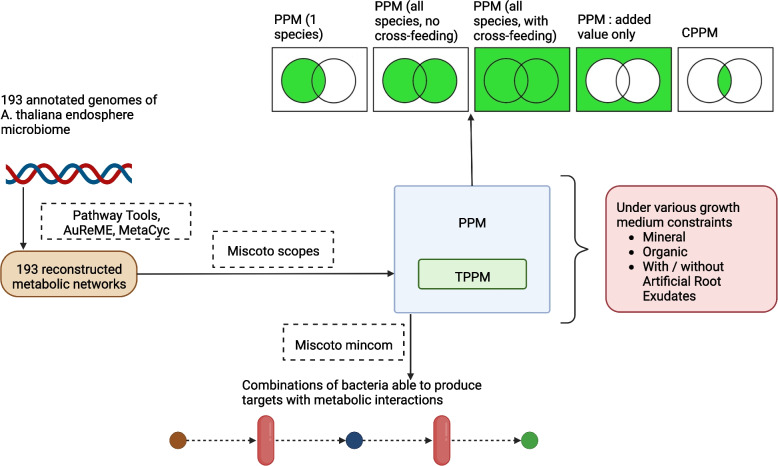
Genome sequence data processing and metric acquisition. Our analysis relies on reconstructed metabolic networks, one per genome, for which PPM and TPPM production were computed, under several nutritional constraints. PPM can also be computed for communities of several cooperating GEMs: in that case, the part of the PPM and TPPM producible only by the community (“added value”) is also returned. Last, minimal combinations of GEMs able to produce as many TPPM as possible were computed

Table 1 lists the specific vocabulary and definitions used in this study. For a summary of genome sequence data processing and metrics acquisition, see Fig. 1.

**Table 1:** Description of the metrics used. Here, the term “community” means either the *whole community* or a random subsample of strains (SynCom). The acronyms in bold in the table are used throughout the text

Metric	Description
**Nutritional constraint**	Available nutrients on which a GEM can rely on (i.e., the initial reactants of the whole network). Nutritional constraints are modeled with simulated growth media. In this paper, an “**unconstrained**” GEM represents its metabolic potential, i.e., all the metabolites it encodes and can theoretically produce.
Predicted Producible Metabolites (**PPM**)	The list (number and composition) of all metabolites predicted to be producible by one or several GEMs simultaneously (also referred to as a **meta-GEM**), under a nutritional constraint or in the absence of a constraint. This metric is used to summarize the **unconstrained and constrained (by available nutrients) metabolism inferred from genomes**.
Core Predicted Producible Metabolites (**CPPM**)	The part (number and composition) of a community PPM which is individually producible by each GEM individually in a set of GEMs.
Targeted Predicted Producible Metabolites (**TPPM**)	A set of 30 metabolites on which part of this study is focused. Their ability to be produced by one or several GEMs is analyzed (number and composition), under a nutritional constraint or in the absence of a constraint.
Community added value	The part of the PPM (number and composition) of several GEMs which is only producible by metabolic interactions within a community (i.e., not producible by a single GEM).
Average phylogenetic distance	The average of all pairwise phylogenetic distances between pairs of strains in a synthetic subsample of strains (**SynCom**). The whole community also has an average phylogenetic distance.

### Genome data

We used 193 annotated genomes [41]⁠ belonging to a culture collection of bacteria isolated from *A. thaliana* roots grown in the Cologne Agricultural Soil (Germany) and representing a taxonomically diverse core set of bacteria of the host plant [41]⁠. Annotated genomes [41, 42]⁠ were downloaded from the At-SPHERE database (http://www.at-sphere.com/). Both the taxonomy and phylogeny of the whole set of genomes [41]⁠ were used. The phylogenetic tree was inferred by maximum likelihood [41]⁠ from a multi-alignment of 31 bacterial AMPHORA [43]⁠ genes obtained with Clustal Omega v1.2.1 [44]⁠ passed into FastTree v2.1 [45]⁠. The genomes were sequenced on a HiSeq2500 (Ilumina Inc, USA) and were considered as high-quality drafts genomes (see Bai et al. [41]). Paired-end reads were trimmed using Trimmomatic v0.33 [46]. Genomes were assembled using A5 [47] and SOAPdenovo [48]. In all cases, the assembly with the smaller number of contigs was selected. Functional annotation was carried out using Prokka v1.11 [49] and the SEED subsystem, using the RAST server API [50]. Data assessing the quality of assembly (N50, N90, total length, number of contigs, number of reads) are available as supplementary data no. 3 and no. 4 in Bai et al. [41]. Elsewhere, a graphical summary of this genomic information is provided in Figure S1. We also checked the quality of the annotation of genomes with Busco v5 [51]. Overall, most of the core genes registered in the Busco database were identified in the genomes (i.e., in almost all the genomes, more than 95% of the searched Busco genes were predicted, Supplementary Figure S1C).

### Reference database

The reference used to link genome annotation to metabolism was the MetaCyc database, a collection of organism-specific Pathway/Genome Databases (PGDBs). MetaCyc contains ~2 500 metabolic pathways from many organisms [52]⁠. Two criteria motivated the choice of this database: first, it is manually curated; second, our tools, which are designed for the reconstruction of the metabolic network (*mpwt* and *AuReMe*) [53, 54] are pre-configured to work with this curated database.

### Reconstruction of the metabolic networks (GEMs)

Metabolic networks of each genome were simulated with genome-scale metabolic models (GEMs) reconstructed with an automated command-line version of PathwayTools [55–57]⁠ using the mpwt program of the metage2metabo tool suite [52]⁠, then converted in padmet and sbml format with AuReMe and padmet-utils [54]⁠. GEMs in *sbml* format were parsed with the Python lxml package when needed. All GEMs were drafts, used without gap-filling or manual curation. These steps are usually required to improve the quality of a GEM [58]⁠, but are likely to introduce false positives, particularly in the case of poorly known organisms, thereby masking potential metabolic dependencies. Consequently, we chose to rely on drafts of GEMs, i.e., we chose false negatives (due to flaws in genome annotation) over false positives.

### Genomes and GEM metrics

In order to detect patterns between strains and metabolism, a set of metrics (the full definitions are listed in Table 1 above) were used and applied to a single GEM (i.e., single strains) and to random combinations (“SynComs”) comprising two to 20 GEMs. The Python API of Miscoto scopes [59]⁠ was used to compute all the predicted producible metabolites, (PPM) for a single GEM, SynComs, and the whole community under simulated nutritional constraints. AuReMe reports were parsed to record constraint-free PPM. Phylogenetic distances were computed based on the phylogenetic tree with the Python package *ete3* [60]⁠. Genome sizes were available in the annotation data. For each SynCom, the core predicted producible metabolites (CPPM) were also computed using sets in Python 3 and AuReMe and Miscoto scopes outputs (Fig. 1). The added value of the full community was also computed with Python sets.

### Targeted predicted producible metabolites (TPPM)

We studied the production capacity of a single GEM and of the whole community (meta-GEM, where all GEMs can leak and exchange any compound) to produce TPPM. The production of TPPM by a GEM can be computed under various nutritional constraints with simulated growth media (see the dedicated section below). We focused on 30 TPPM in this study: 17 amino acids (serine, alanine, and glutamic acid were excluded because they were present in the artificial root exudates, which were part of the growth media), eight B vitamins (thiamine diphosphate, riboflavin, nicotinate, (R)-pantothenate, pyridoxine, biotin, tetrahydro-folate, adenosylcobalamin), and five phytohormones (auxin, salicylic acid, abscisic acid, ethylene, jasmonic acid). Amino acids were chosen for their fundamental, ubiquitous biological importance. Vitamins were chosen according to their importance in metabolism. Phyto-hormones were chosen with respect to the root-associated trait of the microbial community under study. In addition, according to the reference database, these metabolite biosynthesis pathways and the genes encoding them are relatively well-known, easily predictable, and are expected in the studied taxa, thus reducing the risk of misses in the genome assembly, annotation errors, and false negatives in the reconstructed metabolic networks. When under a nutritional constraint (see below), TPPM production was computed for each GEM with the Python API of Miscoto scopes [59]⁠. In the absence of a nutritional constraint, TPPM production was assessed with their absence/presence in AuReMe reports.

### Modeling nutritional constraints (growth media)

Nutritional constraints were established by simulating different growth media. We used two online resources to choose the growth media: MetaCyc (previously mentioned), which contains few growth media with their detailed list of nutrients, and the KOMODO database [61]⁠, which contains a huge number of growth media, together with their composition, and their MetaCyc IDs. We modeled nine growth media (five poorly nutritive with mainly mineral nutrients and four highly nutritive, with more carbon sources, Table 2), to which a common list of cofactors [62]⁠ was added. Each medium has an “artificial root exudate” enriched version (giving a total of 22 media, Table 2), based on Baudoin et al. [63]⁠. Growth media, except rich media, were chosen based on two conditions: (1) none of the TPPM should be included in a medium’s nutrients and (2) the nutrients should not contain any “mixture compounds,” such as tryptone or yeast extract, whose composition is not described in MetaCyc. However, rich media did not fully meet these conditions because they contain vitamins. Among the rich media, LB broth (Lennox) contains tryptone and yeast extract. All the growth media were used to investigate a range of nutritional constraints on PPM, but some were not used for the analysis of the production of TPPM with which they were incompatible (Table 2).

**Table 2:** Summary of the growth media used for nutritional constraints. Since rich media always contain some TPPM, the set of growth media studied varies with the analysis: TPPM were either analyzed all at once, without vitamins, or only phytohormones, depending on the content of the medium concerned. Details on the composition of the media can be found on gitlab

Growth media	Type	Contains TPPM	With mixture	Analyzed TPPM
M63	Poor	No	No	All
M9				
Mineral medium				
Hydrogen oxidizing				
MBM				
Basal	Rich	Vitamins		Amino acids, phyto-hormones
phb pyruvate				
MMJS				
LB-lennox enriched		Vitamins + amino acids	Tryptone (amino acids) + yeast extract	Phyto-hormones

### Putative GEM combinations for metabolic interactions

For each nutritional constraint (i.e., each medium), Miscoto mincom [59]⁠ (a version implemented into the metage2metabo [53]⁠ package) was used with the whole microbial community as input (GEMs in *sbml* format). This tool uses answer set programming, a declarative approach oriented toward combinatorial problem-solving [64, 65]⁠. It rapidly identified all the simplest combinations (called “solutions”) of GEMs able to produce as many specified TPPM as possible under growth constraints. More complex solutions (with more GEMs) are ignored. TPPM were either all the aforementioned compounds, only amino acids, only vitamins, or only phytohormones, in accordance with the aforementioned condition “no TPPM in the growth media” (Table 2). Splitting TPPM was also important because results are strongly TPPM-dependent: for example, GEMs with an essential, but rare reaction for the production of a given TPPM will be very frequently represented, potentially masking alternative possible combinations of other TPPM. The results of each run were stored in a *json* format.

### Quasi-Poisson GLMs

The correlations between the number of PPM/CPPM and SynCom size, genome size, and phylogenetic distance were computed (in the absence of a nutritional constraint) on random SynComs. First, for each number of members (variable “SynCom size”) between 2 and 20 (with a step of 1), 500 SynComs were built by randomly picking strains from the initial pool of 193 strains, without replacement. First, independence between explanatory variables was assessed (Figure S2D). Then, two generalized linear models (GLMs) were built based on a quasi-Poisson distribution (to counter overdispersion of the response metrics, Figure S2, C1 to C4), modeling the response of the number of PPM and CPPM of SynComs according to average phylogenetic distance, average genome size, and SynCom size. A supplemental polynomial (degree 2) term of phylogenetic distance was added to model the curve of the response metrics:

$$\textrm{Y}\sim \textrm{P}\left(\upmu \textrm{i},\uptheta \right)$$

where *i* is a SynCom, *Y* is the number of PPM or CPPM it contains, *P* its average phylogenetic distance, *G* its average genome size, and *S* its number of strains (i.e., GEMS). The maximum SynCom size to include in the model was 12 strains. This size was chosen because of its position at the start of the plateau reached by all the metric values or variance (Figure S2, B1 to B4). This was also the size at which bigger Syncoms started to show no significant difference from one SynCom size to the next when tested with many bootstrapped subsamples (for each SynCom size: 500 iterations of 50 strains each, one-sided Wilcoxon, Mann-Whitney tests, Figure S3). The model residuals were slightly biased and non-normal, caused by the uneven spreads of values in different-sized SynComs (Figures S4 and S5). Since R2 are not automatically returned with the models we used, they were computed with the following formula: 1—residual deviance/null deviance (which are available in the models’ R summaries). The added value of SynCom’s PPM was disregarded for this analysis, because it was computed with Miscoto, which works only under an applied growth constraint.

### Other statistical analyses

Tests of the effect of taxonomy on metric distribution and of the growth media on the number of PPM were performed using non-parametric tests (Wilcoxon and Mann-Whitney rank-sum tests). Corresponding effect sizes were computed with Cliff’s delta method. Principal Coordinate Analysis (PCoA) was performed on Jaccard distance matrices, with the *pcoa* and *vegdist* functions of the R packages ape and Vegan [66]⁠. PERMANOVA was performed on the distance matrices with the Adonis function associated with a multivariate analog of Levene’s test for homogeneity of variances (PERMDISP2 procedure), with the *betadisper* function of the same package. The growth media dendrogram was built by hierarchical clustering with the R base hclust function (with the default “complete” method), after computation of Bray-Curtis distances (with the *vegdist* function) based on the composition of the corresponding whole community PPM (qualitatively, i.e., which compounds are producible under which nutritional constraint, by how many GEMs). Tests involving a taxonomic effect excluded Bacteroidetes and Firmicutes phyla because of their small sample sizes (4 and 7 strains, respectively). The significance threshold was set at 0.01.

### Scripting

Data acquisition and links between tools inputs and outputs (Fig. 1) were organized using homemade Python 3 scripts. All Miscoto outputs were stored in *json* format, and the relevant data they contained (PPM and TPPM, number of genomes producing a TPPM under a given nutritional constraint, etc.) were parsed and stored as *csv* tables. Figures and data analysis were performed with R 4 with the ggplot2 package [67]⁠ and Python 3 with the matplotlib and seaborn packages. Scripts and data are available at https://gitlab.com/mataivic/article_metabolic_modelling_thaliana_microbiome.

## Results

### A link between PPM composition and phylogeny at strain level resolution

**Fig. 2:**
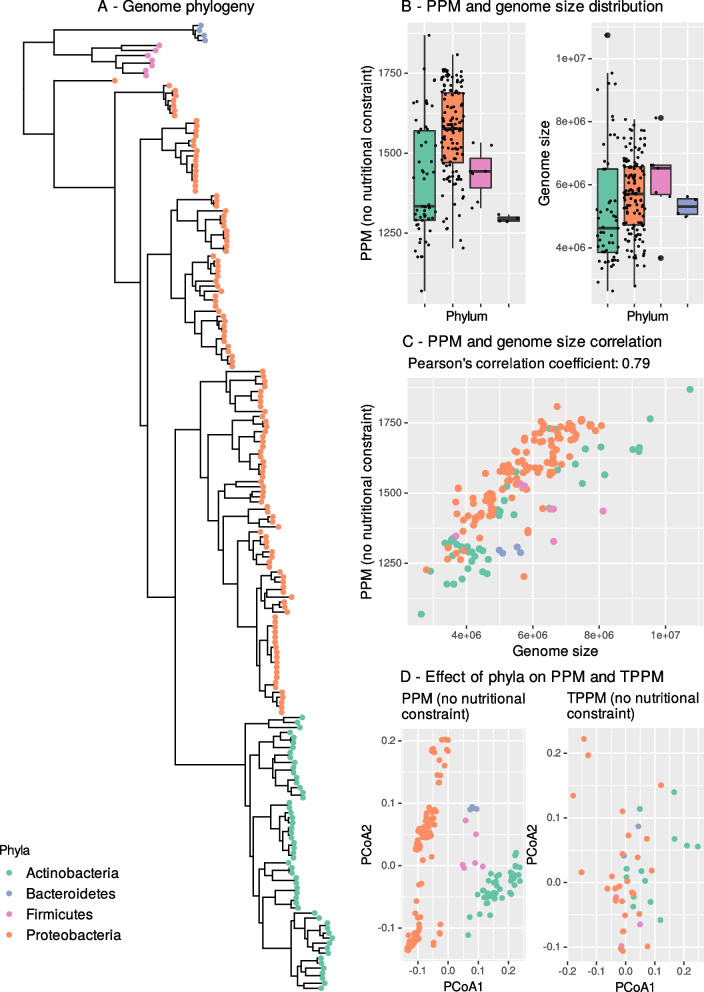
Description of the 193 genomes collected from *A. thaliana* root microbiota **A** Phylogenetic tree (maximum likelihood from a multi-alignment of AMPHORA genes in Clustal Omega). **B** Boxplots displaying the quantitative effect of phyla on genome size and on the distribution of the number of PPM. **C** Dot plots of the number of PPM and TPPM as a function of genome size. **D** PCoA displaying the qualitative effect of phyla on metabolite production (i.e., which compounds are produced by which taxa). Across the panels, colors match genome phyla

We first tested how phylogeny structured the distributions of the different metrics under the hypothesis that metabolic functions derived from unconstrained GEMs’ differ among phylogeny groups (H1). Unconstrained GEMs corresponded to the situation where all the putative genes carried by a genome are considered as functioning (Table 1). Only Actinobacteria and Proteobacteria distributions were statistically tested because the number of strains was insufficient for other phyla. Smaller genome sizes and fewer PPM were observed in Actinobacteria than in Proteobacteria (Fig. 2B, *p*=0.0038 and *p* < 0.0001, with effect sizes of −0.27 and −0.48). The bigger the genome, the bigger the number of PPM and TPPM (Fig. 2C). Bacteroidetes in the culture collection (*n*=4) displayed small genomes and small numbers of PPM, while the number of PPM and the size of the genomes of Firmicutes (*n*=7) were similar to those of the other phyla. Differentiation in PPM composition was also detected among phyla based on PERMANOVAs (*p* < 0.001, *R*^2^=0.213, *p* (permdisp) = 0.005) with well-separated groups (including Bacteroidetes and Firmicutes) observed on PCoA (Fig. 2D). When only considering the 30 selected TPPM, this effect remained significant (*p*<0.001, *R*^2^=0.175, *p* (permdisp) = 0.2325), but between-group differentiation was reduced (Fig. 2D, Figure S6D). The same patterns were observed at the class level (Figure S6). This suggests strong metabolic differentiation between phyla at the whole GEM scale, but a more conserved metabolism at the TPPM scale.

### SynCom PPM under unconstrained metabolism are more diverse than that of single strains and rapidly reach saturation

We extended the previous single-strain GEM approach to random SynComs to analyze the metabolic capacities (number of PPM and CPPM, without nutritional constraint) of merged GEMs (i.e., fully cooperative with all possible metabolic exchanges, H2). The correlations of the number of PPM and CPPM with SynCom size (*n*=2 to 20 members on plots, *n*=2 to 12 in GLMs), mean genome size, and mean phylogenetic distance were explored. The three explanatory variables were significantly correlated with both the number of PPM (*R*^2^=0.86, diagnostic plots in Figure S4) and CPPM (*R*^2^=0.82, diagnostic plots in Figure S5).

**Fig. 3:**
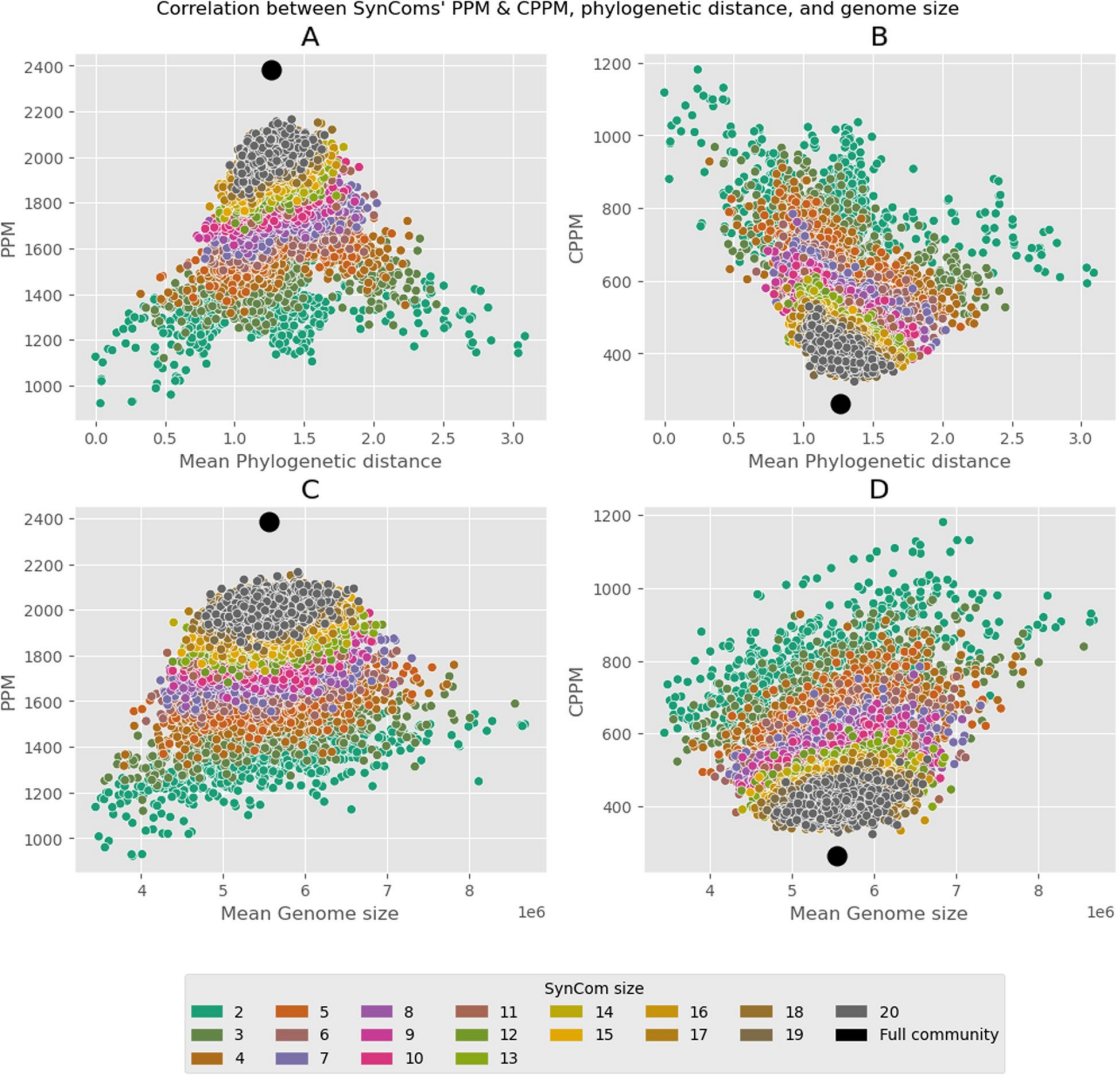
Correlations between explanatory metrics (mean phylogenetic distance (**A**, **B**) and mean genome size (**C**, **D**)) and response metrics: number of PPM (**A**, **C**) and CPPM (**B**, **D**). These correlations were used for the quasi-Poisson GLM models. In each panel, the black dot corresponds to the value for the whole community (193 genomes). The number of PPM increased rapidly with SynCom size, mean phylogenetic distance, and mean genome size, then started plateauing slowly towards the whole community value (see also Figure S7, notably panels B1 to B4). The number of CPPM was negatively correlated with SynCom size and mean phylogenetic distance, and positively correlated with mean genome size

The size of the SynComs was positively correlated with their number of PPM (coefficient=0.36, *p* < 2e−16) and negatively correlated with their number of CPPM (coefficient=−0.65, *p* < 0.001), but this effect was more pronounced in small SynComs. Notably, data from SynComs with many strains largely overlapped, plateauing at about 2000 producible metabolites and 400 core metabolites. Increasing SynCom to 193 strains resulted in increasingly fewer variations in SynComs, until the full community was reached with a PPM of 2 383 and a CPPM of 263. Interestingly, increasing the size of the SynComs rapidly returned numbers of PPM and CPPM close to the whole microbial collection (Fig. 3) likely due to metabolic redundancy. Roughly, SynComs composed of more than ~12 GEMs displayed numbers of PPM and CPPM closer to the values of the whole 193-member community than the values of the smallest SynComs (Fig. 3).

**Fig. 4:**
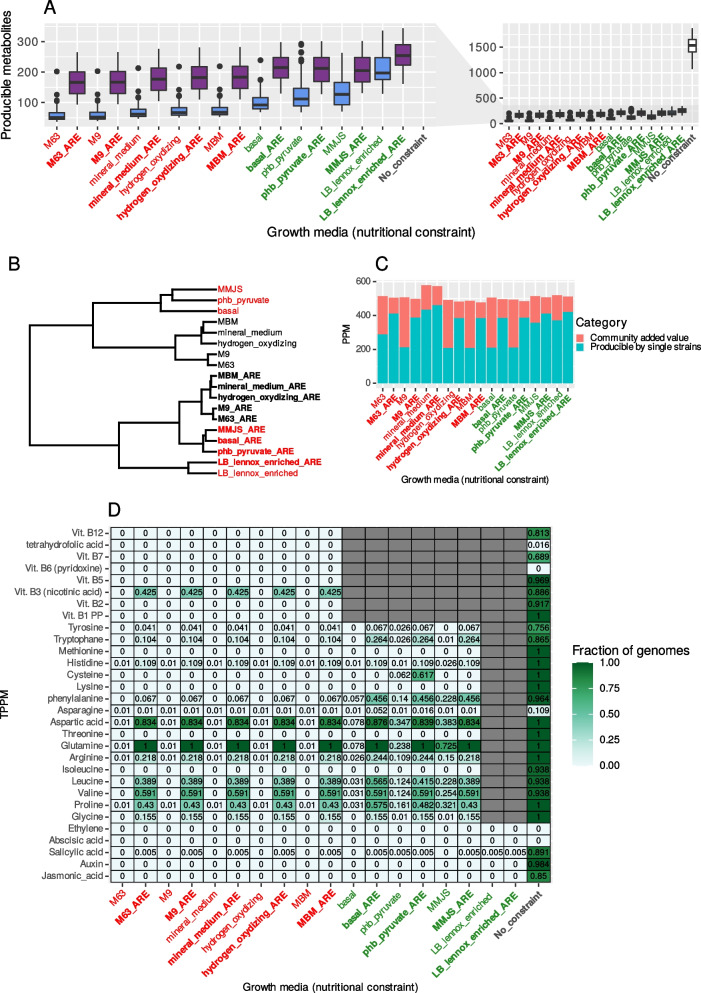
Effect of nutritional constraints on PPM and TPPM. **A** Boxplots of the number of PPM per GEM according to the growth medium, compared to no constraints. **B** Hierarchical clustering (“complete” method, Bray-Curtis distances) of growth media according to the composition of the PPM of the whole community (without community-added value). **C** Number of PPM of the complete community according to the growth media, with the value added by metabolic cross-feeding. **D** TPPM production per growth medium. Decimal numbers indicate the fraction of GEMs capable of producing the compound. Gray cells correspond to growth media already containing the TPPM, which are thus irrelevant. On all axes of the figures, poor media are labeled in black, rich media in red, ARE-enriched media in bold, and standard media in plain text

Phylogenetic distance was positively correlated with SynComs PPM (polynomial coefficients=1.32 and −1.83, *p* < 0.001) and negatively correlated SynComs CPPM (polynomial coefficients=−4.37 and 1.2, *p* < 0.001, Fig. 3A, B). However, PPM reached a peak at a phylogenetic distance of ~1.3 for small SynComs, then decreased (Figs. 3A and 4A). This decrease turned into a plateau when SynCom increased in size (Fig. 3A). CPPM among GEMs first decreased, then reached a plateau. This highlighted increasingly diverse metabolism and increasingly less shared metabolism among strains. Most SynComs, particularly big SynComs, were concentrated around this phylogenetic distance rather than spread equally along all possible distances (Fig. 3 and S4, A1 to A4). This corresponds to the values of most combinations of Proteobacteria and Actinobacteria (Figure S7 A to B) and is explained by the fact that these two phyla were the most frequent in the dataset.

Genome sizes were positively correlated with both PPM (coefficient=0.23, *p* < 0.001) and CPPM (coefficient=0.46, *p* < 0.001, Fig. 3C, D). Thus, genome sizes compensated for the negative effect of phylogenetic distance on metabolic redundancy. At a fixed phylogenetic distance, SynComs with bigger genomes displayed both higher metabolic diversity and a bigger core metabolism than SynComs with smaller genomes (Figure S7 C1&C2). SynComs were also aggregated around an optimal value linked to the corresponding optimal phylogenetic distance and the taxonomic composition of SynComs.

Our results validated that GEM combination from phylogenetically distant strains promotes metabolic diversity, restricts metabolic redundancy, and confers additional metabolic capabilities than single-strain GEMs. Beyond these anticipated results, our results also demonstrated that most meta-GEMs were aggregated around the same mean phylogenetic distance, that a few GEMs were sufficient to approximate whole community metabolism, and that big genomes increased both metabolic diversity and redundancy

### Number and composition of PPM and TPPM are modulated by nutritional constraints

In this analysis, we predicted shifts in GEMs metabolism depending on whether nutritional constraints were applied or not to the model (exemplified by the composition of the simulated growth media, Table 1, H3). “Poor media” refers to severe nutritonal constraints (i.e., with mainly mineral nutrients), while “rich media” refers to more permissive constraints (i.e., with more carbon sources, see the “Methods” section).

At the scale of a single GEM, PPM under nutritional constraints were dramatically reduced compared to PPM of unconstrained GEMs (all *p* < 0.001 with Holm correction, Fig. 4A, right plot). PPM under poor media were significantly fewer in number than PPM under rich media (*p* < 0.001, Cliff’s delta effect size = −0.8978 without ARE and *p* < 0.001, Cliff’s delta effect size = −0.5102 with ARE, Fig. 4A). Supplementing media with compounds that artificially mimicked the exudate composition of plant roots (ARE) was predicted to significantly increase the number of PPM compared to non-supplemented media (green vs. orange in Fig. 4A, Table 3, *p* < 0.001). Notably, the composition of PPM was more similar (i.e., similar sets of producible compounds) across media containing ARE, irrespective of whether the original media were poor or rich (Fig. 4B). Hence, the addition of ARE is predicted to unlock the production of the same metabolites across media. Most of the GEMs have the potential to produce most of the TPPM in the absence of nutritional constraints (27 out of 30 being producible by a single GEM in the full dataset) but cannot complete the entire pathways under most of the nutritional constraints, Fig. 4D). The supplementation of growth media with ARE was predicted to increase the number of TPPM producible by single GEM (Fig. 4D).

**Table 3:** Wilcoxon rank-sum tests on the effect of ARE on the number of PPM

Media (with and without ARE comparison)	Mann-Whitney ***p*** value	Effect size (Cliffs’ delta)
M63	*p* < 0.001*	−0.9786
M9	*p* < 0.001*	−0.9787
Mineral medium	*p* < 0.001*	−0.9788
Hydrogen oxidizing	*p* < 0.001*	−0.9787
MBM	*p* < 0.001*	−0.9788
Basal	*p* < 0.001*	−0.9723
phb_pyruvate	*p* < 0.001*	−0.785
MMJS	*p* < 0.001*	−0.7081
LB lennox enriched	*p* < 0.001*	−0.523

At the whole community scale (i.e., all 193 genomes), the added value provided by a metabolic exchange between all GEMs increased the number of community PPM to similar values regardless of the nutritional constraints (Fig. 4C). Depending on the type of medium (poor/rich) and the absence/presence of ARE, the community-added value increased the number of PPM from 22 to 140% (Fig. 4C) of the number of PPM of the community without metabolic exchange. Thus, GEM functioning is limited by available nutrients that determine which reactions can be activated, but metabolic exchanges between all GEMs of a community can largely compensate for growth constraints, whether they are severe or not.

### The simplest SynComs are predicted to produce TPPM through metabolic exchanges

After exploring the capacity of the whole community to compensate for nutritional constraints, we explored how smaller assemblages can avoid the same constraints. We used “Miscoto mincom” [59], an answer set programming solver designed to automatically find (under a simulated nutritional constraint) all the simplest (i.e., smallest) combinations of GEMs that complete the metabolic pathways to produce the 30 specified TPPM (H4). Combinations of GEMs are considered as a meta-GEM when an incomplete pathway in a single GEM can be completed by another, thanks to exchange of intermediate products.

**Fig. 5:**
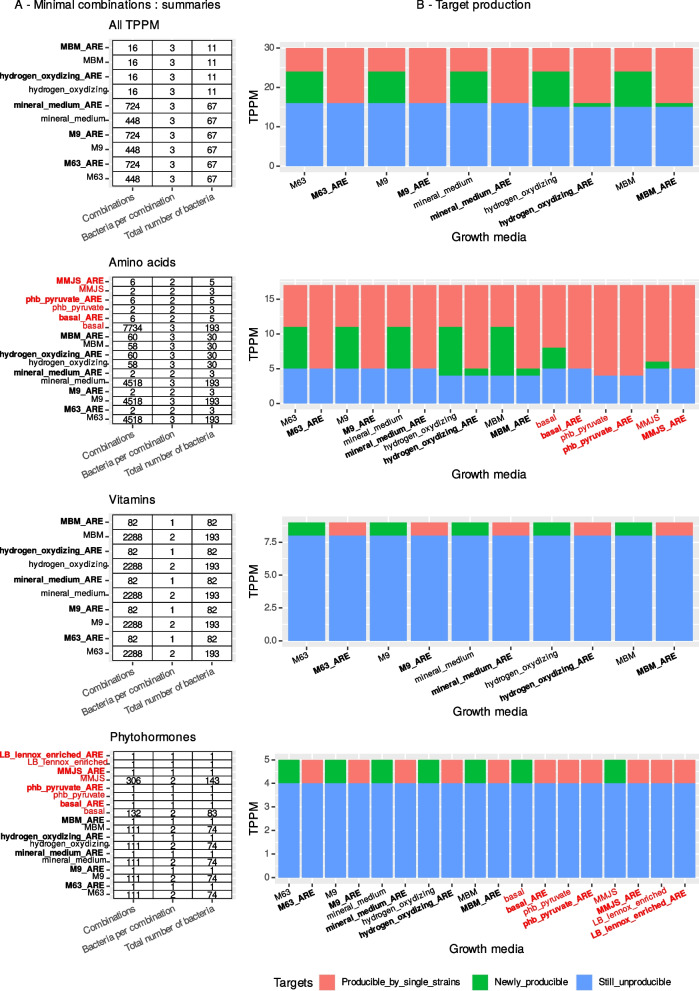
Minimal combinations of GEMs needed to produce TPPM. **A** Summary of the number of combinations of GEMs able to produce TPPM, for all TPPM together, amino acids only, vitamins only, and phytohormones only. **B** Details of producible and unproducible TPPM with single GEM capacities (red) and added values of combinations with metabolic completions (green). TPPM and nutritional constraints are ordered in the same way as in panel **A**. Results are given for each nutritional constraint (black: poor, red: rich), without (plain) or with (bold) ARE. The list of nutritional constraints varies depending on the TPPM considered, in accordance with the no-TPPM-in-media condition (i.e., growth media containing TPPM in their composition were excluded when necessary). On all axes in all the figures, poor media are labeled in black, rich media in red, ARE-enriched media in bold, and standard media in plain text

There were many possible—always small—combinations of two or three GEMs sufficient to produce some of the the TPPM. The sets of combinations were also largely identical from one medium to another (Fig. 5A), suggesting an important effect of identical nutrients among growth media and shared reactions among GEMs. The total number of GEMs involved in combinations varied depending on the TPPM included in the analysis. For example, 67 GEMs were returned when all TPPM were considered in poor media (11 in rich media), while the whole set of 193 GEMs (for a few media) was returned when only amino acids or vitamins were considered (Fig. 5A).

Globally, rich media (amino-acid and phytohormone TPPM) produced very few combinations, meaning they allow more GEMs to produce TPPM without predictable mandatory metabolic dependencies or cooperation (Fig. 5A). For example, there were less than 10 minimal combinations of two different GEMs predicted to produce 12 amino acids under rich media, while simulations using poor media returned dozens to thousands of combinations of two or three GEMs. The addition of ARE also reduced the number of simplest combinations (for example from 2288 to 82 for rich media with vitamins as TPPM), except for the situation “all TPPM + poor media” (Fig. 5A, first row). In such a situation, the ARE-enriched poor media contained more combinations (724 for ARE-enriched poor media, 448 for standard poor media, for 14 producible TPPM).

The combinations of GEMs increased the number of TPPM compared to single-GEM capacities under several growth constraints (Fig. 5B). In poor media, six to seven supplemental amino acids were predicted to be producible by thousands of combinations of two or three GEMs compared to individual GEMs (often six amino acids). Interestingly, for rich media, each amino acid was predicted to be producible by at least one GEM, but no GEM could produce by itself all the 17 targeted amino acids, resulting in a few combinations of strains predicted to exchange end products instead of intermediate metabolites (Fig. 5B). Among vitamins and phytohormones, only nicotinic acid (vitamin B3) and salicylic acid pathways were predicted as complete, with metabolic exchanges required between two GEMs under severe nutritional constraints.

**Fig. 6:**
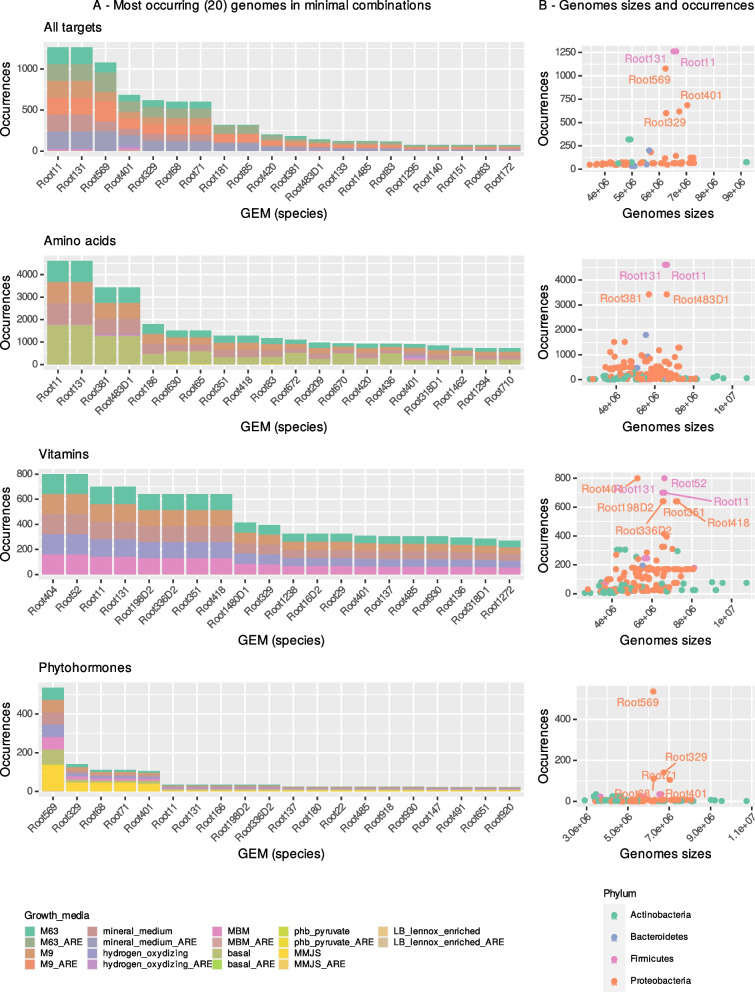
Frequencies of GEMs in minimal combinations needed to produce TPPM. **A** The 20 most frequently occurring GEMs in all possible minimal combinations for all TPPM, for amino acids only, for vitamins only, and for phytohormones only. **B** Occurrences of GEMs in minimal combinations as a function of the size of their genome. Text-annotated dots indicate the most frequently occurring GEMs. No relation was found between the size of a genome and its frequency in the combinations

The frequency of GEMs in the solutions was highly varied with a few GEMs occurring in several hundred solutions (Fig. 6A). The other GEMs were much less frequent with only a few occurrences. Thus, for all TPPM, most solutions can be aggregated with a set of seven GEMs (Supplementary Table S1, seven first rows), belonging to strains of the phyla Proteobacteria and, surprisingly, Firmicutes (despite being very underrepresented in the dataset). When TPPM are split according to the category, the majority of solutions can be aggregated with a set of 15 GEMs (Supplementary Table S1). We found no clear correlations between the frequency of a GEM in the solutions and the size of its corresponding genome (Fig. 6B). In fact, the high frequency of these particular GEMs was driven by their strong contribution to the production of a few particular TPPM (Fig. 6A), notably with the case of salicylic acid (Figure S8). In MetaCyc, the bacterial salicylic acid pathway is composed of two reactions depending on the availability of chorismate (which has a longer pathway), but other reactions that take place outside this pathway are also recorded in the database. The first reaction is encoded into five GEMs only, which are part of the aforementioned seven main GEMs. The second reaction is encoded into 47 GEMs. Most of the combinations for the completion of this pathway of salicylic acid are then built with these GEMs. We noted that 47 GEMs differ from the total of 74 GEMs returned by the solver, which highlights the production of salicylic acid by other means than the pathways described above (as described in Lefevere et al. [68]⁠ in plants and Mishra and Baek [69]⁠ in plants and bacteria). One GEM was remarkable in that it was the only one predicted to encode the complete salicylic acid pathway: the *Pseudomonas* identified as “Root569” and could putatively produce salicylic acid when growth conditions are optimal.

Hence, the answer set programming solver predicts that many small combinations of GEMs could compensate for diverse nutritional constraints by exchanging metabolites, unlocking the production of several TPPM inaccessible by a single GEM.

## Discussion

### Fundamental niche signature in GEMs

The analysis of the PPM of the 193 GEMs of the *A. thaliana* culture collection demonstrated that phylogenetically related bacterial species share more similar metabolism than distant species. Our first hypothesis (H1) was thus validated in line with the literature [70]⁠. Previous research attempted to predict ecological traits from genomic and metabolic information [33, 71]⁠, highlighting differentiation between taxonomic groups and hierarchical conservation within groups [72]⁠. However, finding fundamental niche signatures in GEMs remains a challenging task [28]⁠, and complementary trait-based approaches were used to determine ecological attributes or correlate overlapping niches with phylogeny [71, 73]⁠⁠.

### Phylogenetic distance, similarity and complementarity, antagonism, and cooperation in SynComs

SynComs allowed more diverse metabolism when they contain phylogenetically distant strains, our second hypothesis (H2) was thus validated, along with many unpredicted results (discussed below). Several studies used metrics similar to ours and obtained comparable results [17, 74–76], highlighting a correlation between metabolic similarity/dissimilarity and phylogenetic distance. Phylogenetically distant bacteria can be predicted to have less metabolic resource overlap and a higher potential to cooperate [76]⁠, while phylogenetically closer taxa are expected to compete [17]⁠. Based on phylogenetic similarity and dissimilarity, other studies attempted to go further and interpret whether niche differentiation or habitat filtering was at play. Results are conflicting and research is currently far from a consensus. For example, some authors found that species in the gut microbiome tend to co-occur more frequently with their competitors, thus highlighting habitat filtering [18]⁠, while others showed that increased phylogenetic relatedness was correlated with competitive exclusion among bacterivorous protist species [77]⁠, thus favoring niche differentiation.

Rather than favoring niche differentiation or habitat filtering, SynComs unconstrained metabolism question the aggregation of most SynComs around a putative optimal phylogenetic distance, along with a metabolic diversity peak for smaller SynComs (Fig. 3A). The peak is probably due to the taxonomic composition of SynComs with a high phylogenetic distance. These SynComs all incorporate a Firmicutes or a Bacteroidetes (Figure S7 A&B), which both display lower metabolic capacities than the other phyla, causing an inevitable decrease in PPM compared to other combinations. However, the aggregation of SynComs around the same phylogenetic distance is more interesting. Closely related bacteria have similar needs and hence face strong competition for resources [15]⁠ despite many cross-feeding opportunities. Conversely, very distant bacteria avoid competition, i.e., are adapted to colonize different niches, and their needs only overlap to a limited extent. The observed optimal phylogenetic distance in our results could be driven by the taxonomic composition of the dataset (mostly composed of Proteobacteria and Actinobacteria), and the composition is itself partially determined by the host plant [78, 79]⁠. More importantly, this optimum can be interpreted as a trade-off between metabolic similarity and dissimilarity. Optimum niche overlap would limit competition for resources among closely related species while being sufficient to promote exchanges of metabolites. However, the existence of microbial communities at both ends of the competitive-cooperation spectrum has also been highlighted, mostly dependent on the environment (soil, free-living, or host-associated environments) [80]⁠.

### Metabolism is nutritional-constraint dependent

Sets of constraints were modeled using growth media with different compositions. Our third working hypothesis (H3) stating that the available nutrients have a significant impact on the metabolism of bacteria was thus validated.

GEMs displayed low metabolic capabilities under severe growth constraints, alleviated by ARE in line with the nature of the genome collection used, i.e., isolated from *A. thaliana* roots. This result suggests that exudates from plant roots are important determinants of the range of producible metabolites in the root microbiome. The effect of ARE was particularly visible for TPPM: the same set of TPPM was produced with the addition of ARE, regardless of the growth medium. This could either reflect the fact that our TPPM are core compounds on which most bacteria rely and are expected to metabolize. TPPM are hypothesized to create similar metabolic patterns among GEMs, activated by ARE.

These findings highlight the importance of nutrient availability for the metabolic profile of the bacterial community [81]⁠. However, there is currently no consensus on the effects of all nutrients on microbial communities. For example, despite nitrogen being a strong predictor of metabolism, its enrichment has different impacts on the diversity and composition of soil microbiota [82]⁠. Root exudates are known to modify microbial communities, but only the effect of a few compounds was recently elucidated [83–85]⁠.

The marked difference between unconstrained and constrained metabolism could also be due to the difficulty to correctly model an environment using simulated nutritional constraints. Indeed, some TPPM were impossible to reproduce in any medium, despite full completion rates within several GEMs (such as auxin and B12 vitamin). However, it could also reflect the fact that most growth media are not adapted to all organisms, many of which are known to be difficult to grow in vitro [86]⁠. Hence, metabolic cooperation would be especially relevant with such microorganisms. Reverse ecology has been attempted to avoid in vitro culture problems, for example, by computing the set of nutrients required by a metabolic network to produce biomass [64, 75, 76, 87]⁠. Such approaches allowed the computation of overlapped and differentiated growth requirements of several organisms, thereby advancing our knowledge of the ecological niche and metabolic interactions.

### Metabolic dependencies are predicted to be major drivers of microbial community structure

Metabolic exchanges were found to be essential to improve the metabolic capacities of GEMs, both at the scale of the whole community and at the scale of a combination of a few GEMs, thus validating our fourth hypothesis (H4). However, we did not expect such big differences between poor and rich media, nor the counterintuitive effect of ARE on very poor media with many TPPM.

### Metabolic exchanges depend on nutritional constraints and compensate for severe growth constraints

Rich growth media and ARE unlocked more PPM and TPPM for single GEMs, with little metabolic cooperation required (Fig. 5). Indeed, growth constraints are likely weaker in rich media since most nutrients are available, thereby unlocking many reactions and their associated metabolic pathways. Reciprocally under poor media, i.e., severe growth constraints, single GEMs were not self-sufficient and more metabolic exchanges were required to produce some TPPM. This was reflected by the marked difference in the number of possible combinations of GEMs able to produce TPPM. However, when considering all TPPM at once in very poor media (M9, M63, mineral medium), supplementation with ARE had a different effect and increased the number of predicted combinations of GEMs (Fig. 5A), which was surprising as they are supposed to improve single GEM autonomy. We concluded that under very severe nutritional constraints, an extremely small subset of GEMs can produce intermediate products and cooperate. In this case, ARE unlocked enough chemical reactions in other GEMs to compensate for the constraints, making several of them self-sufficient for some TPPM. Hence, there was no need for cross-feeding of intermediate compounds, but no GEM was self-sufficient for all the TPPM, suggesting a possible exchange of finished products (hence more combinations). The combined effect of the availability of nutrients in the soil and nutrients secreted by the host might then be a powerful driver of metabolic interactions. In support of this hypothesis, Klitgord and Segré [88]⁠ found that there is always a way to predict a growth medium inducing metabolic interactions between pairs of seven species. However, they failed to predict a viable medium for individual species, again highlighting the importance of cooperation. Finally, at the scale of the whole community, the major compensation predicted by cooperation between all GEMs (Fig. 4C) also underlines the importance of metabolic cooperation to counter strong nutritional constraints.

### Minimal combinations of GEMs reflect functional redundancy for the targeted compounds

The results allowed us to predict putative cooperation between GEMs able to produce relevant TPPM of the root microbiome (amino acids, vitamins, phytohormones). In most TPPM categories, a number of putative combinations were returned when strong nutritive constraints and were applied with or without ARE. These results are in agreement with those obtained by Frioux et al. [59]⁠ and Thommes et al. [89]⁠. The number of solutions predicted echoed recent research which predicted a wide range of metabolites that can be secreted without cost, generating countless cross-feeding opportunities [90]⁠. Even if these results are tightly linked to the TPPM and constraints considered, combinations of bacteria are predicted to be able to co-metabolize to complete core, ubiquitous metabolic pathways. This observation can be interpreted as community-level functional redundancy [59]⁠ and as an insurance of the completion of metabolic processes for nutrition or interaction with host plants under a range of environmental constraints [91]⁠. These putative redundancies in metabolic completions could play a key role in maintaining stability under variable environmental constraints. Beyond these core metabolic functions, it would be interesting to extend the analysis of minimal communities to secondary, more specific metabolism.

The software used, “Miscoto mincom” [59]⁠, worked in a way such that only the most parsimonious solutions are calculated, the simplest combinations of GEMs that fit the applied constraints. However, these putative solutions do not mean more complex combinations do not exist to produce the TPPM. Finally, we did not explore all possible solutions to distinguish mutualistic and unidirectional cross-feeding. One strain could be the final producer of a TPPM by taking advantage of the secretion of another strain, i.e., with no mutualistic exchange, or alternatively, several exchanges may be required. Both interacting behaviors likely coexist, even at the level of a single bacterium, depending on the other bacteria. To date, knowledge is lacking on these behaviors among co-existing bacteria. Other hypotheses concerning the bacterial secretomes may better explain what actually happens in living systems.

### The effect of genome size remains unclear

Bacteria with big genomes are usually considered as generalist species with wider niches [92]⁠. They indeed have a higher unconstrained metabolism (higher PPM) thus likely a higher probability to possess uncommon, important reactions involved in the production of the chosen TPPM. Antagonistic bacteria are also more likely to have larger genomes, linking antagonistic, and generalist strategies [74]⁠. Conversely, bacteria with small genomes are more likely to be involved in metabolic interactions due to their reduced set of reactions [93, 94]. Our results showed that bacteria with larger genomes exhibited both higher metabolic similarity and complementarity than others (Figure S7 C1&C2), pointing to a putative reservoir of functions which compensated for low phylogenetic distances, but without providing many clues about their orientation towards cooperation or antagonism. The absence of any correlation between GEMs’ corresponding genome size and their frequencies in putative metabolic interactions also prevented us from establishing a link between generalist/specialist behavior and cooperation or auxotrophies provider.

### Only a few strains are needed to reach the community potential

As the size of SynComs increased, their metabolism quickly became similar (Fig. 3 and S4). According to these results, the whole community’s unconstrained metabolism can be approximated using only a few dozen GEMs (linked with H2). In addition, most combinations for the production of TPPM involved a reduced set of GEMs, recalling previous studies which identified core microbiota composed of a reduced pool of species [5]⁠ to perform and/or optimize a biological function [95]⁠, analyze the impact of core strains on the whole microbiome [91, 96], or to study host colonization processes [41]⁠. Our results suggest the existence of core functions, echoing other studies reported that the functional stability of the microbiota is maintained regardless of the strains chosen, as long as each functional group is chosen [97]⁠.

## Conclusions and prospects

Metabolic diversity and similarity were detected according to the genome taxonomy. The multi-genome metabolic modeling analyses we performed enabled us to predict functional inter-dependencies and revealed a long-lasting ecological paradigm, a trade-off between competition and cooperation. We also found that putative metabolic interactions are common and constraint-dependent, thereby revealing community-level interlinkages and cooperation that make it possible to buffer nutritional constraints. The large number of interactions underlines the importance of richness and diversity in microbial communities for community-level functioning. Taken together, these results provide clues to the best way to decipher microbial interactions in a microbiota beyond the limits of the set of genomes used in the present study.

In this perspective, deeper and more realistic genome-based modeling approaches based on the cost of exchanges, flux analysis, and the use of continuous nutrient depletion over time could provide a closer look at the community-level genomic toolbox used to respond to constraints and to decipher evolutionary and behavioral responses to these constraints in either fluctuating or constant environments. All approaches leading to putative responses will need to be tested experimentally. With this aim in view, multi-omics data, including secretome [25]⁠, a current research frontier, could be used to overcome the challenges to data interoperability. For instance, single isolates could be cultivated on various growth media, and analysis of their secretome would be a way to test the predictions on PPM. A deeper analysis could involve co-cultures of synthetic communities made of two (or more) isolates, predicted to compete or to be metabolically interdependent by the exchange of compounds. Also, cultures of isolates on a minimal medium enriched with the secretome of other isolates would be an interesting way to study the dependency of an isolate on another while limiting any other interaction, such as competition.

## Supplementary Information

**Additional file 1: Figure S1.** General information about genomic data and genomes annotations of the culture collection used. (A) Distribution of the ratios of N50 over genome length. N50 cannot be directly compared because genomes have various length. Using such a ratio allows a comparison between N50. The closer to 1, the lesser small contigs are needed to cover 50% of the genome. Most genomes have a N50 of approximately 10% of the total genome length, and a few genomes have very high N50. (B) Distribution of the number of contigs in the genomes assemblies. (C) Distribution of the annotation completeness of Busco's core genes. The closer to 100, the more all core genes in Buco's database are present in the annotation. **Figure S2.** In order to fix the maximum Syncom size to inject into Poisson GLMs, we compared every (s, s+1) pair of SynComs’ PPM and CPPM, s being a SynCom size in [2, 20]. For each (s, s+1) pair, 200 pairs of random subsets of size n=50 SynComs were taken, and their PPM and CPPM were tested with Wilcoxon, Mann & Whitney tests. Boxplots of the 200 p values are displayed for all size comparisons and for PPMs (top) and CPPMs (bottom). Red lines are p=0.05 and green crosses are 1st quantiles. SynComs of 12 strains were chosen as a limit because it was the minimum size at which less than 10% of the p values were under 0.05 for PPM and CPPM. **Figure S3.** Description of the 193 genomes collected from A. thaliana root microbiota (A) Phylogenetic tree (maximum likelihood on a multi-alignment of AMPHORA genes). (B) Boxplots displaying the quantitative effect of class on genome sizes and PPM distributions. (C) Plots of the producible metabolites or TPPM as a function of genome sizes. (D) PCoA displaying the qualitative effect of phyla on metabolite production (i.e. which compounds are produced by which taxa). Colours match classes of strains. **Figure S4.** Details of the correlations between explanatory metrics and response metrics for SynComs with two strains only. (A-B) Patterns of SynComs taxonomic composition in the correlation between PPM and CPPM and phylogenetic distance. Proteobacteria and Actinobacteria combinations are responsible for the PPM peak. (C-D) there is also a taxonomic signal among the PPM and CPPM responses to genome size. (E-F) Effect of genome sizes on SynComs' PPM and CPPM. SynComs with a bigger average genome size have both bigger PPMs and CPPMs than SynComs with a smaller average genomes size. For each plot, only SynComs with 2 strains are shown. **Figure S5.** Density plots show that for each SynCom size, most values are concentrated around a narrow range. (B1 to B4) Boxplots showing the reach of a plateau (in terms of values and/or variances) for each metric. Only data for SynComs’ size below the plateaus were kept in the regression models (size 2 to 12 strains). (C1 to C4) distributions of the different metrics, split by SynCom size. (D) phylogenetic distance and genome size are not correlated, making their use as independent variables valid in the quasi-Poisson regression. **Figure S6.** Diagnostic plots of the quasi-poisson GLM modelling the response of PPM in SynComs. **Figure S7.** Diagnostic plots of the quasi-poisson GLM modelling the response of the CPPM in SynComs. **Figure S8.** A schematic view of the salicylic acid biosynthesis pathway. There are only two reactions, possessed respectively by 47 and 6 GEMs, Root569 being the only GEM with a complete pathway. Under not constraining growth media, Root569 has the capacity to produce salicylic acid by itself but lost this ability under severe nutritional constraints. In such cases, the set of strains has to exchange intermediate metabolites to produce salicylic acid. A possible hypothesis is that Root569 is incapable of producing chorismate under severe growth constraints, whereas other strains are. **Table S1.** Taxonomy of the most frequently occurring GEMs in the putative combinations of GEMs permitting TPPM production through metabolic exchanges. The TPPM categories in which each GEM is the most involved are mentioned.

## Data Availability

All the datatsets are available online and all the scripts are available on the GitLab https://gitlab.com/mataivic/article_metabolic_modelling_thaliana_microbiome. Sequenced genomes are available on the At-SPHERE database (http://www.at-sphere.com/)
