# Supplementary material for: Multi-genome metabolic modeling predicts functional inter-dependencies in the Arabidopsis root microbiome
Source: Microbiome. 2022 Dec 9;10:217. doi: 10.1186/s40168-022-01383-z (PMC9733318; doi:10.1186/s40168-022-01383-z)
Supplement: Supplementary file 2 — Additional file 1: Figure S1. General information about genomic data and genomes annotations of the culture collection used. (A) Distribution of the ratios of N50 over genome length. N50 cannot be directly compared because genomes have various length. Using such a ratio allows a comparison between N50. The closer to 1, the lesser small contigs are needed to cover 50% of the genome. Most genomes have a N50 of approximately 10% of the total genome length, and a few genomes have very high N50. (B) Distribution of the number of contigs in the genomes assemblies. (C) Distribution of the annotation completeness of Busco's core genes. The closer to 100, the more all core genes in Buco's database are present in the annotation. Figure S2. In order to fix the maximum Syncom size to inject into Poisson GLMs, we compared every (s, s+1) pair of SynComs’ PPM and CPPM, s being a SynCom size in [2, 20]. For each (s, s+1) pair, 200 pairs of random subsets of size n=50 SynComs were taken, and their PPM and CPPM were tested with Wilcoxon, Mann & Whitney tests. Boxplots of the 200 p values are displayed for all size comparisons and for PPMs (top) and CPPMs (bottom). Red lines are p=0.05 and green crosses are 1st quantiles. SynComs of 12 strains were chosen as a limit because it was the minimum size at which less than 10% of the p values were under 0.05 for PPM and CPPM. Figure S3. Description of the 193 genomes collected from A. thaliana root microbiota (A) Phylogenetic tree (maximum likelihood on a multi-alignment of AMPHORA genes). (B) Boxplots displaying the quantitative effect of class on genome sizes and PPM distributions. (C) Plots of the producible metabolites or TPPM as a function of genome sizes. (D) PCoA displaying the qualitative effect of phyla on metabolite production (i.e. which compounds are produced by which taxa). Colours match classes of strains. Figure S4. Details of the correlations between explanatory metrics and response metrics for SynComs with two strains only. [file 40168_2022_1383_MOESM1_ESM.docx]

Multi-genome metabolic modelling predicts functional inter-dependencies in the *Arabidopsis* root microbiota

Victor Mataigne^1,2^, Nathan Vannier^2^, Philippe Vandenkoornhuyse^1,*^, Stéphane Hacquard^2,*^

1 Université de Rennes 1, CNRS, UMR6553 ECOBIO, Campus Beaulieu, 35000 Rennes, France

2 Max Planck Institute for Plant Breeding Research, 50829 Cologne, Germany

Supplementary information


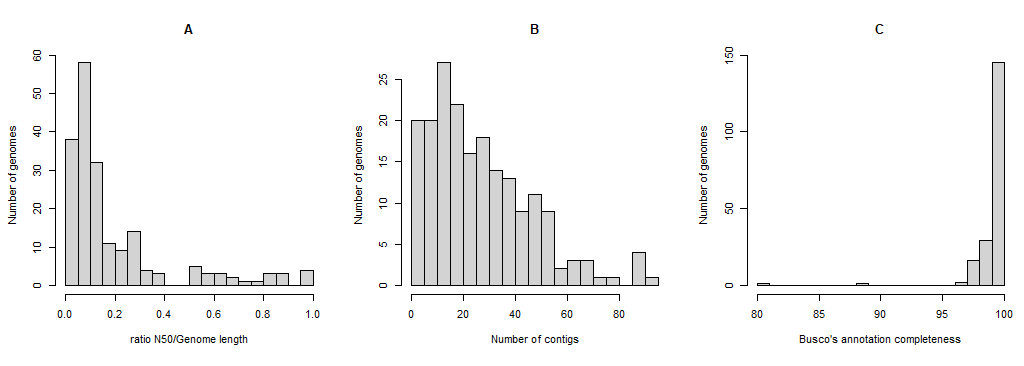
**Figure S1**: General information about genomic data and genomes annotations of the culture collection used. (A) Distribution of the ratios of N50 over genome length. N50 cannot be directly compared because genomes have various length. Using such a ratio allows a comparison between N50. The closer to 1, the lesser small contigs are needed to cover 50% of the genome. Most genomes have a N50 of approximately 10% of the total genome length, and a few genomes have very high N50. (B) Distribution of the number of contigs in the genomes assemblies. (C) Distribution of the annotation completeness of Busco's core genes. The closer to 100, the more all core genes in Buco's database are present in the annotation.


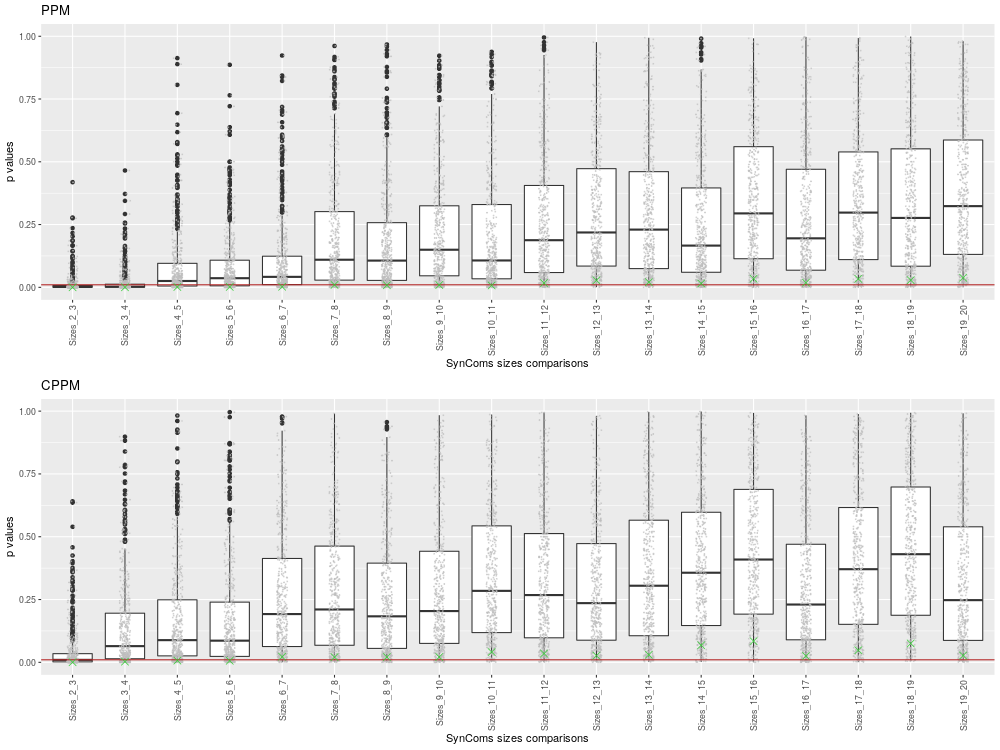

Figure FF**Figure S2**: in order to fix the maximum Syncom size to inject into Poisson GLMs, we compared every (s, s+1) pair of SynComs’ PPM and CPPM, s being a SynCom size in [2,20]. For each (s, s+1) pair, 200 pairs of random subsets of size n=50 SynComs were taken, and their PPM and CPPM were tested with Wilcoxon, Mann & Whitney tests. Boxplots of the 200 p values are displayed for all size comparisons and for PPMs (top) and CPPMs (bottom). Red lines are p=0.05 and green crosses are 1st quantiles. SynComs of 12 strains were chosen as a limit because it was the minimum size at which less than 10% of the p values were under 0.05 for PPM and CPPM.


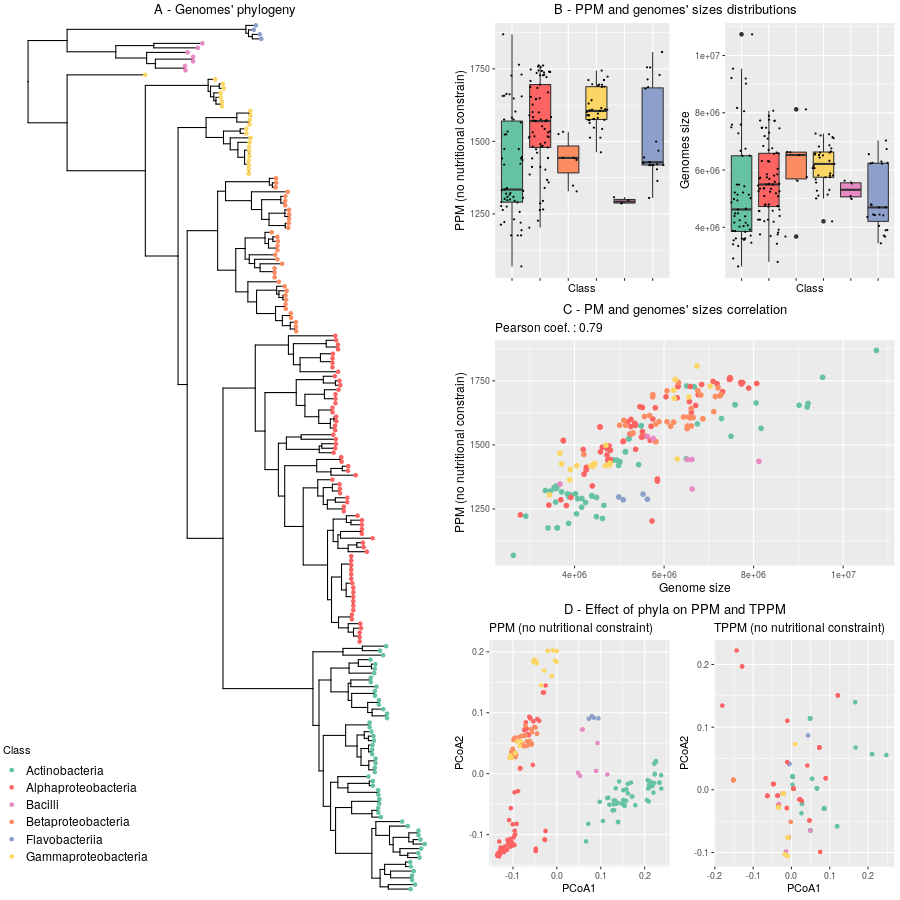

**Figure S3**: description of the 193 genomes collected from *A. thaliana* root microbiota (A) Phylogenetic tree (maximum likelihood on a multi-alignment of AMPHORA genes). (B) Boxplots displaying the quantitative effect of class on genome sizes and PPM distributions. (C) Plots of the producible metabolites or TPPM as a function of genome sizes. (D) PCoA displaying the qualitative effect of phyla on metabolite production (i.e. which compounds are produced by which taxa). Colours match classes of strains.


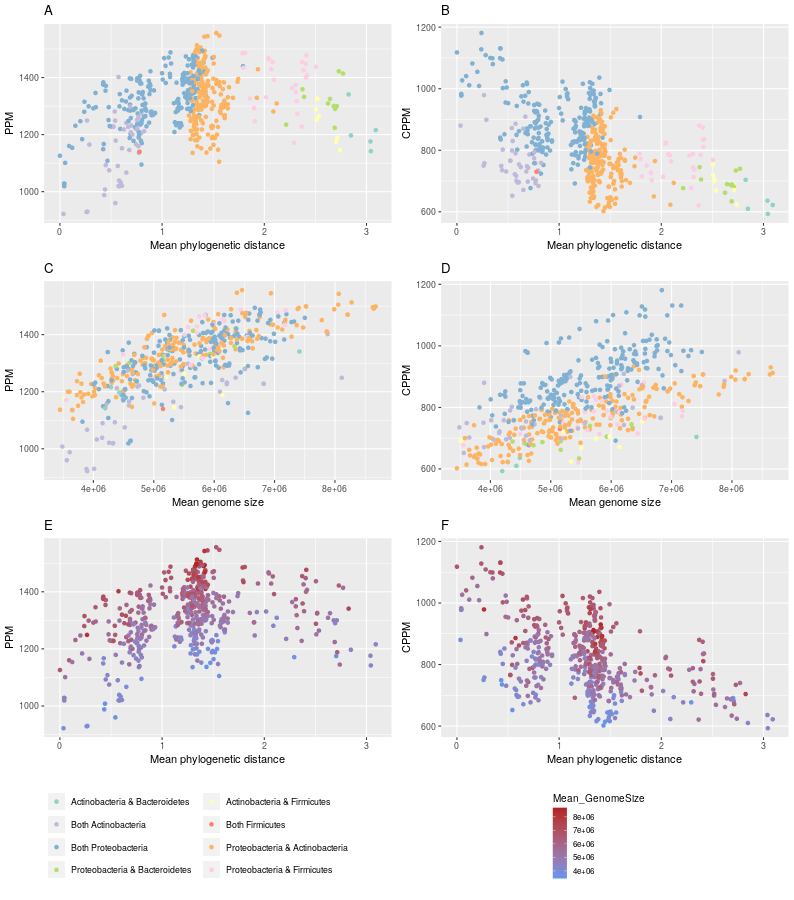
**Figure S4**: details of the correlations between explanatory metrics and response metrics for SynComs with two strains only. (A-B) Patterns of SynComs taxonomic composition in the correlation between PPM and CPPM and phylogenetic distance. Proteobacteria and Actinobacteria combinations are responsible for the PPM peak. (C-D) there is also a taxonomic signal among the PPM and CPPM responses to genome size. (E-F) Effect of genome sizes on SynComs' PPM and CPPM. SynComs with a bigger average genome size have both bigger PPMs and CPPMs than SynComs with a smaller average genomes size. For each plot, only SynComs with 2 strains are shown.


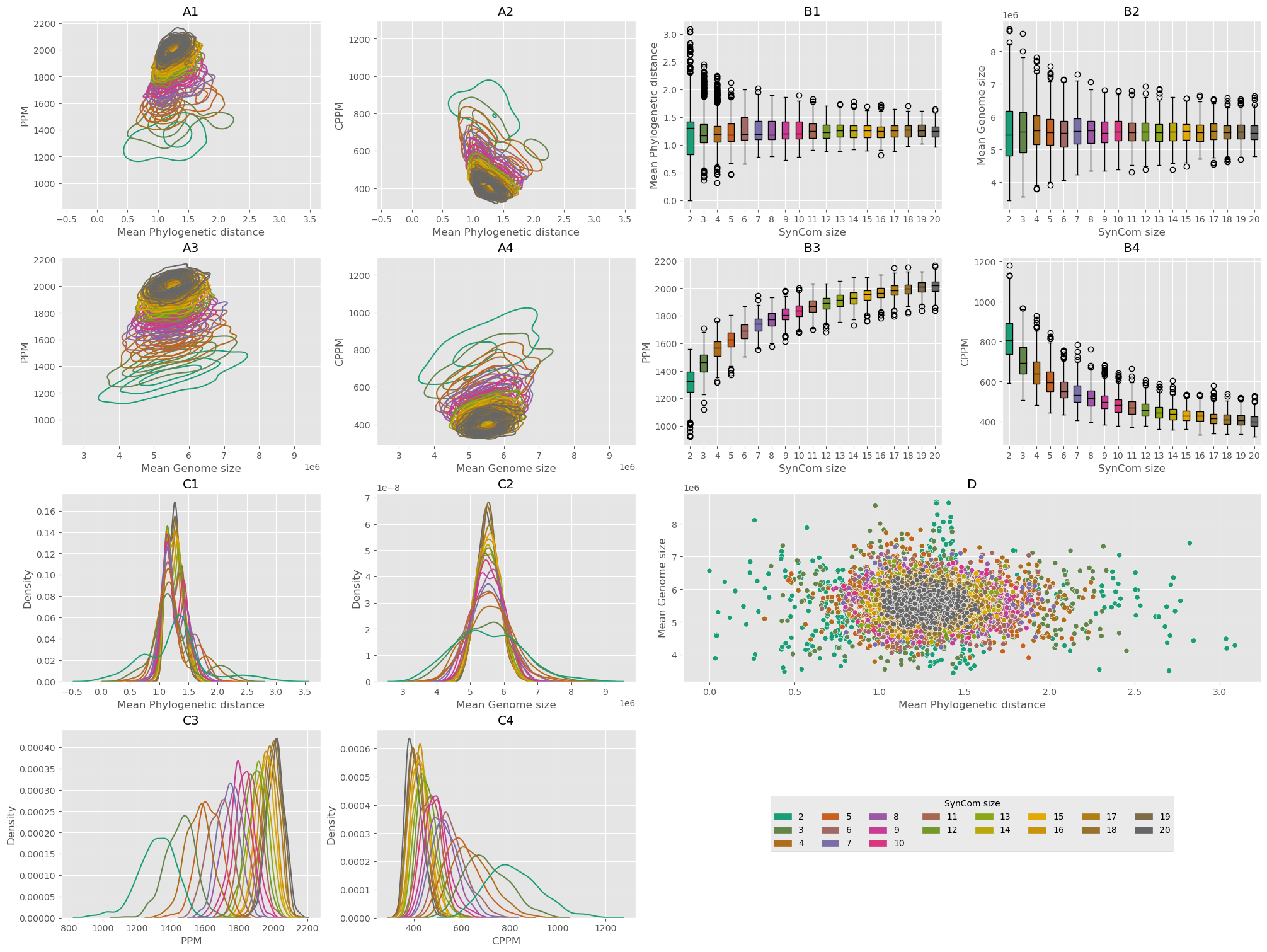

**Figure S5**: density plots show that for each SynCom size, most values are concentrated around a narrow range. (B1 to B4) Boxplots showing the reach of a plateau (in terms of values and/or variances) for each metric. Only data for SynComs’ size below the plateaus were kept in the regression models (size 2 to 12 strains). (C1 to C4) distributions of the different metrics, split by SynCom size. (D) phylogenetic distance and genome size are not correlated, making their use as independent variables valid in the quasi-Poisson regression.


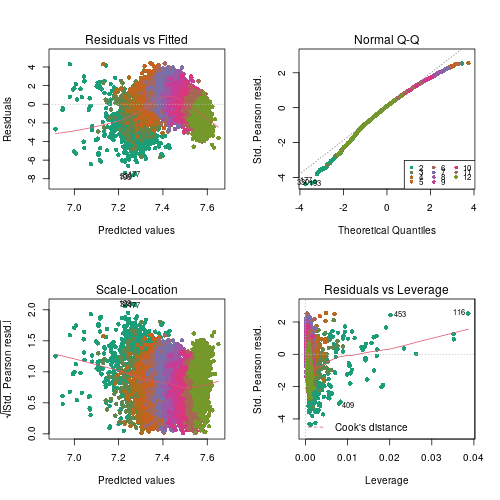

**Figure S6:** diagnostic plots of the quasi-poisson GLM modelling the response of PPM in SynComs.


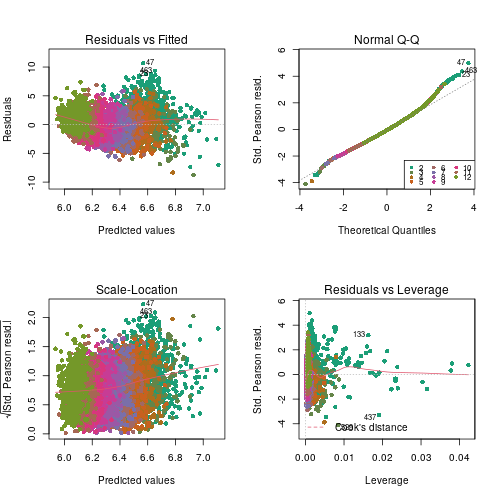

**Figure S7**: diagnostic plots of the quasi-poisson GLM modelling the response of the CPPM in SynComs


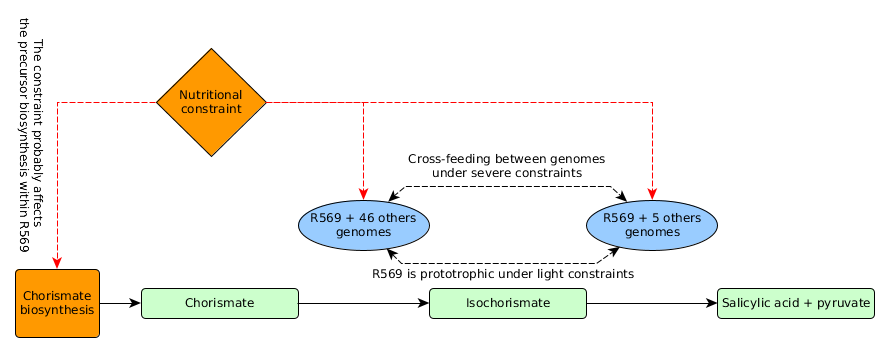

**Figure S8**: a schematic view of the salicylic acid biosynthesis pathway. There are only two reactions, possessed respectively by 47 and 6 GEMs, Root569 being the only GEM with a complete pathway. Under not constraining growth media, Root569 has the capacity to produce salicylic acid by itself but lost this ability under severe nutritional constraints. In such cases, the set of strains has to exchange intermediate metabolites to produce salicylic acid. A possible hypothesis is that Root569 is incapable of producing chorismate under severe growth constraints, whereas other strains are.

**Table S1** : Taxonomy of the most frequently occurring GEMs in the putative combinations of GEMs permitting TPPM production through metabolic exchanges. The TPPM categories in which each GEM is the most involved are mentioned.

| **Identifier** | **Involved TPPM** | **Phylum** | **Class** | **Order** | **Family** | **Genus** |
| --- | --- | --- | --- | --- | --- | --- |
| Root11 | All | Firmicutes | Bacilli | Bacillales | Bacillaceae | Bacillus |
| Root131 | Amino-acids, vitamins | Firmicutes | Bacilli | Bacillales | Bacillaceae | Bacillus |
| Root569 | Phyto-hormones (salicylic acid) | Proteobacteria | Gammaproteobacteria | Pseudomonadales | Pseudomonadaceae | Pseudomonas |
| Root401 | Phyto-hormones (salicylic acid) | Proteobacteria | Gammaproteobacteria | Pseudomonadales | Pseudomonadaceae | Pseudomonas |
| Root329 | Phyto-hormones (salicylic acid) | Proteobacteria | Gammaproteobacteria | Pseudomonadales | Pseudomonadaceae | Pseudomonas |
| Root68 | Phyto-hormones (salicylic acid) | Proteobacteria | Gammaproteobacteria | Pseudomonadales | Pseudomonadaceae | Pseudomonas |
| Root71 | Phyto-hormones (salicylic acid) | Proteobacteria | Gammaproteobacteria | Pseudomonadales | Pseudomonadaceae | Pseudomonas |
| Root381 | Amino-acids | Proteobacteria | Alphaproteobacteria | Rhizobiales | Bradyrhizobiaceae | Bosea |
| Root483D1 | Amino-acids | Proteobacteria | Alphaproteobacteria | Rhizobiales | Bradyrhizobiaceae | Bosea |
| Root404 | Vitamins (B3) | Proteobacteria | Betaproteobacteria | Burkholderiales | Rhizobacter | NA |
| Root52 | Vitamins (B3) | Firmicutes | Bacilli | Bacillales | Paenibacillaceae | Paenibacillus |
| Root198D2 | Vitamins (B3) | Proteobacteria | Betaproteobacteria | Burkholderiales | Oxalobacteraceae | Duganella |
| Root336D2 | Vitamins (B3) | Proteobacteria | Betaproteobacteria | Burkholderiales | Oxalobacteraceae | Duganella |
| Root351 | Vitamins (B3) | Proteobacteria | Betaproteobacteria | Burkholderiales | Oxalobacteraceae | Massilia |
| Root418 | Vitamins (B3) | Proteobacteria | Betaproteobacteria | Burkholderiales | Oxalobacteraceae | Massilia |
